# Anxiety and depression in Charcot-Marie-Tooth disease: data from the Italian CMT national registry

**DOI:** 10.1007/s00415-022-11365-8

**Published:** 2022-09-16

**Authors:** Marta Bellofatto, Alessandro Bertini, Irene Tramacere, Fiore Manganelli, Gian Maria Fabrizi, Angelo Schenone, Lucio Santoro, Tiziana Cavallaro, Marina Grandis, Stefano C. Previtali, Isabella Allegri, Luca Padua, Costanza Pazzaglia, Daniela Calabrese, Paola Saveri, Aldo Quattrone, Paola Valentino, Stefano Tozza, Luca Gentile, Massimo Russo, Anna Mazzeo, Giuseppe Vita, Sylvie Piacentini, Chiara Pisciotta, Davide Pareyson, for the Italian CMT Network, Giulia Schirinzi, Maria Montesano, Sara Nuzzo, Francesca Oggiano, Chiara Gemelli, Marina Scarlato, Emanuele Spina, Maria Longo

**Affiliations:** 1 Unità di Malattie Neurodegenerative e Metaboliche Rare, Dipartimento di Neuroscienze Cliniche, Fondazione IRCCS Istituto Neurologico Carlo Besta, Via Celoria 11, 20133 Milan, Italy; 2 Dipartimento Gestionale di Ricerca e Sviluppo Clinico, Direzione Scientifica, Fondazione IRCCS Istituto Neurologico Carlo Besta, 20133 Milan, Italy; 3 Dipartimento di Neuroscienze, Scienze Riproduttive ed Odontostomatologiche, Università Federico II di Napoli, 80131 Naples, Italy; 4 Dipartimento di Neuroscienze, Biomedicina e Movimento, Università di Verona, 37126 Verona, Italy; 5 Dipartimento di Neuroscienze, Riabilitazione, Oftalmologia, Genetica e Scienze Materno-Infantili, Università di Genova, 16132 Genoa, Italy; 6 IRCCS Ospedale Policlinico San Martino, 16132 Genoa, GE Italy; 7 INSPE and Division of Neuroscience, IRCCS Ospedale San Raffaele, 20132 Milan, Italy; 8 U.O. Neurologia, A.O. di Parma, 42126 Parma, Italy; 9 Università Cattolica del Sacro Cuore, 00168 Rome, Italy; 10 Fondazione Policlinico Universitario A. Gemelli IRCCS, 00168 Rome, Italy; 11 Centro di Ricerca delle Neuroscienze, IBFM-CNR, Università Magna Grecia di Catanzaro e Unità di Ricerca di Neuroimaging, Germaneto, 88100 Catanzaro, Italy; 12 Dipartimento di Scienze Mediche, Università Magna Grecia, 88100 Catanzaro, Italy; 13 Unità di Neurologia e Malattie Neuromuscolari, Dipartimento di Medicina Clinica e Sperimentale, Università di Messina, 98124 Messina, Italy; 14 Unità di Neuropsicologia, Dipartimento di Neuroscienze Cliniche, Fondazione IRCCS Istituto Neurologico Carlo Besta, 20133 Milan, Italy

**Keywords:** Depression, Anxiety, HADS, HMSN (Charcot-Marie-Tooth), Peripheral neuropathies

## Abstract

**Background:**

There is little information about neuropsychiatric comorbidities in Charcot-Marie-Tooth disease (CMT). We assessed frequency of anxiety, depression, and general distress in CMT.

**Methods:**

We administered online the Hospital Anxiety-Depression Scale (HADS) to CMT patients of the Italian registry and controls. HADS-A and HADS-D scores ≥ 11 defined the presence of anxiety/depression and HADS total score (HADS-T) ≥ 22 of general distress. We analysed correlation with disease severity and clinical characteristics, use of anxiolytics/antidepressants and analgesic/anti-inflammatory drugs.

**Results:**

We collected data from 252 CMT patients (137 females) and 56 controls. CMT patient scores for anxiety (mean ± standard deviation, 6.7 ± 4.8), depression (4.5 ± 4.0), and general distress (11.5 ± 8.1) did not differ from controls and the Italian population. However, compared to controls, the percentages of subjects with depression (10% vs 2%) and general distress (14% vs 4%) were significantly higher in CMT patients. We found no association between HADS scores and disease duration or CMT type. Patients with general distress showed more severe disease and higher rate of positive sensory symptoms. Depressed patients also had more severe disease. Nineteen percent of CMT patients took antidepressants/anxiolytics (12% daily) and 70% analgesic/anti-inflammatory drugs. Patients with anxiety, depression, and distress reported higher consumption of anxiolytics/antidepressants. About 50% of patients with depression and/or general distress did not receive any specific pharmacological treatment.

**Conclusions:**

An appreciable proportion of CMT patients shows general distress and depression. Both correlated with disease severity and consumption of antidepressants/anxiolytics, suggesting that the disease itself is contributing to general distress and depression.

**Supplementary Information:**

The online version contains supplementary material available at 10.1007/s00415-022-11365-8.

## Introduction

Charcot-Marie-Tooth disease (CMT) is the most common inherited neuromuscular disorder and affects motor and sensory peripheral nerves [1, 2]. Most CMT patients develop distal limb muscle weakness, distal sensory loss, foot deformities, and decreased deep tendon reflexes. Disease severity is usually assessed using the composite scale Charcot-Marie-Tooth Examination/Neuropathy Score (CMTES and CMTNS) [3].

Mood disorders are among the least studied comorbidities in CMT. Previous researches have reported that severe depression affects 40–50% of patients with other chronic neurologic disorders such as multiple sclerosis, Parkinson’s disease, Huntington’s chorea [4–6]. In contrast, prevalence of depression in amyotrophic lateral sclerosis and other neuromuscular disorder appears to be surprisingly low [7–9]. As far as CMT is concerned, large systematic studies about frequency and management of neuropsychiatric disorders are not available. This issue may be particularly important since untreated mood disorders may worsen patients’ own perception of health and neurological status. The few published studies have investigated mostly the presence of depression using the Beck Depression Inventory (BDI) in the context of wider analyses of disability and quality of life [10–12].

Our study is the first one to focus on both depression and anxiety disorders in a large series of CMT patients by employing the Hospital Anxiety and Depression Scale (HADS). This validated scale is one of the most commonly used for assessing anxiety and depression among patients in a general hospital setting [13, 14] and has been widely used to measure general distress, anxiety, and depression in many neurological disorders, such as multiple sclerosis [15] and Parkinson’s disease [16].

We performed a large case–control study among CMT patients adhering to the Italian CMT Registry and their relatives/friends, to assess frequency of anxiety and depression disorders in CMT patients, and their correlation with CMT course and pharmacological treatment.

## Methods

We built a National CMT Registry [17] (www.registronmd.it) in collaboration with the Italian patients’ associations, including ACMT-Rete, and Telethon-Italy Foundation. It is a dual registry where the patient registers herself/himself, chooses a reference centre among nine spread all over Italy (Fondazione IRCCS Istituto Neurologico Carlo Besta of Milan, IRCCS Ospedale San Raffaele of Milan, Universities of Genoa, Verona, Parma, Rome, Naples, Catanzaro and Messina) where the attending clinician, in an ad hoc visit, collects a minimal data set of information and administers clinical scales (CMTES/CMTNS) [3]. All Registry records are reviewed and data confirmed by local medical officers and validated by the Registry curator.

Healthy controls were recruited among friends and unaffected relatives of CMT participants, matched as much as possible for gender and age. The study was approved by the Ethics Committee of the Fondazione IRCCS Istituto Neurologico Carlo Besta, Milan (no. 52/2016 Date: April 2, 2014), and then by the Ethics Committees of all the other eight participating centres. It conforms with World Medical Association Declaration of Helsinki. A written informed consent was obtained from all participants to the Registry and an online informed consent was obtained from all those completing the questionnaires (patients and controls). The recruitment lasted 3 years (2015–2017). Both patients and controls were asked to fill online the validated Italian version of the HADS [14], a self-assessed questionnaire composed of fourteen statements, seven exploring the presence of anxiety-related symptoms (HADS-A scale) and seven the presence of depression symptoms (HADS-D scale), making up a total score (HADS-T) indicative of general distress. Each statement is rated by a four-point (0–3) system (range 0–42, were 0 is no general distress at all and 42 the worst score). We set the cut-off score at ≥ 11 for both the HADS-A and HADS-D to define moderate-to-severe anxiety/depression, and at ≥ 22 for the whole HADS-T scale to indicate moderate-to-severe general distress, based on the results reported by Singer et al., where ≥ 11 and ≥ 22 where the cut-off values with the best positive predictive values, with high specificity and a reasonable sensibility [14–16, 18].

We compared data from CMT patients with those of healthy controls and the Italian population available from the literature [19].

### Statistical analysis

A description of participant characteristics at baseline was provided in terms of absolute numbers and percentages for categorical data and means with standard deviations (SDs) and medians for continuous data. We then used the Mann–Whitney *U* test, Fisher’s exact test, and Spearman’s Rank-Order Correlation to analyse associations between HADS scores and type of participants (CMT patients vs controls), gender, age, disease duration, disease severity (CMT examination score, CMTES), walking ability and/or use of orthotics, hand disability, sensory symptoms, and medication use (antidepressant/anxiolytic drugs; analgesic/anti-inflammatory drugs).

Throughout the statistical analysis, the significance level was set at 0.05 (significant: < 0.05).

### Data sharing and accessibility

Data that support the findings of this study are deposited in an ad hoc repository and are available from the senior author (DP) to be shared anonymously on request from any qualified investigator. All data relevant to the study are included in the article or uploaded as online supplemental information.

## Results

The HADS questionnaire was filled by 252 CMT patients and 56 controls (20 friends and 36 unaffected relatives). Demographic and clinical data, and HADS scores of CMT participants and controls are summarized in Table 1 and in Supplementary Table 1. The age of controls and CMT patients were similar, whereas there was a slight, although not significant, predominance of females in the CMT group as compared to controls. Among the 252 CMT patients, 117 (46.4%) had a diagnosis of CMT1A; the other most frequent CMT types were CMTX1 (*n* = 21, 8.3%), CMT1B (*n* = 18, 7.1%), CMT2I/CMT2J (*n* = 14, 5.6%), CMT2A (*n* = 8, 3.2%), CMT4C (*n* = 5, 1.9%). HADS scores for anxiety, depression and general distress did not differ between patients and controls and the normal Italian population (Table 1) [19]. The scores for anxiety and general distress were significantly higher in females in both patients and controls; no difference was found between genders for the HADS-D scores (Table 1).

**Table 1:** Demography, disease severity, and HADS scores of CMT patients, healthy controls and the Italian population [19]

	Patients			Controls			Italian population		
	Females (*n* = 137)	Males (*n* = 115)	Total (*n* = 252)	Females (*n* = 24)	Males (*n* = 32)	Total (*n* = 56)	Females (*n* = 829)	Males (*n* = 770)	Total (*n* = 1599)
Age (mean ± SD)	46.6 ± 14.4	47.5 ± 13.8	47.1 ± 13.1	46.2 ± 12.7	43.9 ± 12.5	44.9 ± 12.6	–	–	–
CMTES (mean ± SD, median)	8.0 ± 5.1, 7	8.4 ± 5.1, 8	8.2 ± 5.1, 8	–	–	–	–	–	–
Anxiety HADS-A Score (mean ± SD, median)	7.8 ± 4.8, 7*	5.9 ± 4.6, 5*	6.9 ± 4.8, 6	7.8 ± 4.5, 7.5*	4.5 ± 3.4, 3.5*	5.9 ± 4.2, 5	8.0 ± 4.3	7.0 ± 4.5	7.6 ± 4.4
Depression HADS-D Score (mean ± SD, median)	5.0 ± 4.1, 4	4.2 ± 3.8, 3	4.5 ± 4.0, 3	4.2 ± 3.3, 3	3.0 ± 2.4, 2.5	3.5 ± 2.9, 3	5.4 ± 3.9	5.5 ± 4.2	5.4 ± 4.0
General distress HADS-T Score (mean ± SD, median)	12.8 ± 8.2, 11*	10.2 ± 7.8, 8*	11.5 ± 8.1, 10	12.0 ± 6.6, 12.5*	7.5 ± 5.3, 6.5*	9.4 ± 6.2, 8	13.4 ± 7.5	12.5 ± 8.1	13.0 ± 7.8

*A* anxiety, *CMTES* Charcot-Marie-Tooth Examination Score, *D* depression, *F* females, *HADS* Hospital Anxiety and Depression Scale, *M* males, *SD* standard deviation, *T* total *HADS-A and HADS-T scores were significantly higher in females than males both in the CMT (*p* = 0.001 and *p* = 0.007, respectively) and in the control group (*p* = 0.006 and *p* = 0.014, respectively). There were no significant differences between CMT and controls and between CMT and the Italian population in terms of HADS-A, HADS-D and HADS-T scores

Overall, 19.2% of CMT patients for whom the information was available (*n* = 45/234) took antidepressants and/or anxiolytics medications (Supplementary Table 1), with 12% (*n* = 28) taking them every day. Seventy percent of them made use of analgesic/anti-inflammatory drugs (*n* = 164/234) (Supplementary Table 1), 17% (*n* = 40) more than once per week.

Table 2 reports the distribution of patients and controls with HADS scores above or below the cut-off thresholds selected for identifying moderate-to-severe anxiety/depression/general distress versus no or mild anxiety/depression/general distress. We found moderate-to-severe anxiety, depression, and general distress (HADS-A and HADS-D scores ≥ 11 and HADS-T ≥ 22) in 25.4%, 10.3% and 14.2% of CMT patients, respectively. As compared to CMT, the percentages of subjects with moderate-to-severe depression and general distress were significantly lower in controls (1.8% for depression, *p* = 0.038; 3.5%, for general distress, *p* = 0.025). The percentage of subjects with moderate-to-severe anxiety was rather high also in controls (14.4%) and the difference with CMT was not significant (*p* = 0.083). We could not compare such percentages with the normal Italian population as the information on the percentages of anxious, depressed and distressed subjects was not available from the reference [19] and to the best of our knowledge there is no other paper dealing with HADS normative values in the Italian population.

**Table 2:** Distribution of CMT patients and controls with HADS scores above or below the cut-off thresholds for moderate-to-severe anxiety/depression/general distress versus no or mild anxiety/depression/general distress

	Patients (*n* = 252)	Controls (*n* = 56)	p*
Anxiety, *n* (%)			
HADS-A < 11	188 (74.6%)	48 (85.6%)	0.083
HADS-A ≥ 11 moderate-to-severe anxiety	64 (25.4%)	8 (14.4%)	
Depression, *n* (%)			
HADS-D < 11	226 (89.7%)	55 (98.2%)	**0.038**
HADS-D ≥ 11 moderate-to-severe depression	26 (10.3%)	1 (1.8%)	
General distress, *n* (%)			
HADS-T < 22	216 (85.8%)	54 (96.4%)	**0.025**
HADS-T ≥ 22 moderate-to-severe distress	36 (14.2%)	2 (3.5%)	

*A* anxiety, *D* depression, *HADS* Hospital Anxiety and Depression Scale, *T* total

*p values based on Fisher's exact test. Significant differences are reported in bold

We did not observed differences in the levels of anxiety/depression/general distress between the different CMT subtypes (Table 3).

**Table 3:** Comparison between CMT1A and other CMT subtypes for HADS-A, HADS-D, and HADS-T scores

	CMT1A (*n* = 117)	Other CMTs (*n* = 135)
Anxiety HADS-A score (mean ± SD, median)*	6.9 ± 4.7, 6	6.8 ± 4.8, 6
Depression HADS-D score (mean ± SD, median)*	4.4 ± 4.0, 3	4.8 ± 3.9, 4
General Distress HADS-Total score (mean ± SD, median)*	11.4 ± 8.1, 9	11.7 ± 8.1, 10
Anxiety: HADS-A score ≥ 11, *n* (%)**	28 (24%)	36 (27%)
Depression: HADS-D score ≥ 11, *n* (%)**	12 (10%)	14 (10%)
General Distress: HADS-Total score ≥ 22, *n* (%)**	16 (14%)	20 (15%)

*A* anxiety, *D* depression, *HADS* Hospital Anxiety and Depression Scale, *T* total

There were no significant differences for these items between CMT1A and other CMTs. We used the Mann–Whitney *U* test (*) or the Fisher’s exact test (**), as appropriate. *p* values are not shown

We then evaluated differences in terms of clinical characteristics between patients with and without anxiety, depression, and general distress (as defined by scores above/below the cut-off values) [18] (Table 4). We found that CMT patients with moderate-to-severe depression and general distress had more severe disease as assessed by the CMTES. There were no differences in terms of age and disease duration (data not shown). Compared to patients with normal scores, patients with anxiety and general distress complained more frequently of positive sensory symptoms. The use of analgesic/anti-inflammatory drugs was significantly higher among patients reporting positive sensory symptoms (80%) as compared to subjects without positive sensory symptoms (63%, *p* = 0.005).

**Table 4:** HADS scores by clinical characteristics of the CMT population

	Anxiety		Depression		General distress	
	HADS-A < 11 (*n* = 188)	HADS-A ≥ 11 (*n* = 64)	HADS-D < 11 (*n* = 226)	HADS-D ≥ 11 (*n* = 26)	HADS-T < 22 (*n* = 216)	HADS-T ≥ 22 (*n* = 36)
Gender	Females 97 (51%)	Females 40 (62%)	Females 119 (53%)	Females 18 (69%)	Females 113 (52%)	Females 24 (67%)
	Males 91 (49%)	Males 24 (38%)	Males 107 (47%)	Males 8 (31%)	Males 103 (48%)	Males 12 (33%)
		(*p* = 0.130)		(*p* = 0.108)		(*p* = 0.109)
CMTES (mean ± SD, median)	8.0 ± 5.0, 7	8.7 ± 5.5, 8 (*p* = 0.342)	**8.2 ± 5.2, 7**	9.6 ± 4.7, 10 (*p* = 0.031)*	**8.0 ± 5.2, 7**	9.3 ± 4.6, 9 (*p* = 0.041)*
Walking difficulties *n* (%)	138 (73%)	50 (78%) (*p* = 0.509)	166 (73%)	22 (84%) (*p* = 0.340)	159 (73%)	29 (81%) (*p* = 0.417)
Orthotics aid *n* (%)	76 (40%) (AFO: 15%)	20 (31%) (*p* = 0.181) (AFO: 15%)	90 (39%) (AFO: 16%)	6 (23%) (*p* = 0.134) (AFO 2%)	86 (39%) (AFO 15%)	10 (27%) (*p* = 0.197) (AFO 14%)
Walking support need *n* (%)	24 (13%)	8 (13%) (*p* = 1)	28 (12%)	4 (15%) (*p* = 0.754)	28 (13%)	4 (11%) (*p* = 1)
Positive sensory symptoms *n* (%)	**66 (35%)**	32 (50%) (*p* = 0.038)**	84 (37%)	14 (53%) (*p* = 0.135)	**78 (36%)**	20 (56%) (*p* = 0.040)**
Difficulties with buttons *n* (%)	107 (57%)	41 (64%) (*p* = 0.378)	129 (57%)	19 (73%) (*p* = 0.142)	123 (57%)	25 (69%) (*p* = 0.200)
Anxiolytics/antidepressants *n* (%)	**25 (14%)**	20 (35%) (*p* = 0.002)**	**34 (16%)**	11 (52%) (*p* < 0.001)**	**31 (15%)**	14 (43%)* (*p* < 0.001)**
Analgesics/anti-inflammatory *n* (%)	119 (68%)	45 (78%) (*p* = 0.363)	148 (68%)	16 (76%) (*p* = 0.670)	138 (68%)	26 (81%) (*p* = 0.450)

*A* anxiety, *CMTES* Charcot-Marie-Tooth Examination Score, *D* depression, *HADS* Hospital Anxiety and Depression Scale, *SD* standard deviation, *T* total

*p* values are reported in bold for significant differences. * = Mann–Whitney *U* test. ** = Fisher’s exact test

Notably, patients with anxiety, depression and general distress reported more frequent use of anxiolytics/antidepressants and analgesic/anti-inflammatory drugs. It is also noteworthy that about one half of patients with anxiety, depression, and/or general distress did not receive any drug treatment for these disturbances. Indeed, only 35% of patients showing anxiety (*n* = 20/58), 52% of depressed patients (*n* = 11/21), and 43% of patients with general distress (*n* = 14/32) reported they were treated with drugs for anxiety/depression.

## Discussion

This is the first study investigating the presence of anxiety, depression and general distress using the HADS tool in a large series of CMT patients (*n* = 252). Even if the mean scores for CMT were similar to those of the control group and the normal Italian population [19], there was a significantly higher rate of CMT patients with depression and general distress as compared to controls.

Although the HADS scale has been previously used in other neuromuscular disorders, such as amyotrophic lateral sclerosis and Duchenne muscular dystrophy, showing the same prevalence of depression than other neuromuscular disorders and the general population [7, 8], it has never been used in large enough samples of CMT subjects. Indeed, only a few studies investigated the presence of depression in CMT, the majority using the BDI, and yield conflicting results [9–12, 20–22]. In a series of 73 CMT patients, the mean BDI score was normal (6.1, normal values 5–9) and the prevalence of current neuropsychiatric disorders (assessed with the Structured Clinical Interview for DSM-IV) was 11%, similar to patients with Duchenne muscular dystrophy and facioscapulohumeral dystrophy [9]. In other studies, researchers administered the BDI to CMT patients in the context of larger analysis [10, 11] focussing on quality of life and found no relationship with clinical examination, but for a slight direct association between depression and referred paresthesias and hypoesthesia [11]. The Minnesota Multiphasic Personality Inventory (MMPI) was within the normal range in a series of 23 CMT patients [23]. Vinci and colleagues evaluated anxiety and depression with the Kellner’ symptoms questionnaire (KSQ) Italian validated version and found no difference between 53 CMT subjects and 53 controls [24]. On the other hand, Ivanovic and colleagues observed that 29% of a series of 45 CMT1A patients had symptoms of depression according to the BDI, which were a cause of worse quality of life [12]. Depression negatively affects quality of life in patients with hereditary neuropathies also according to Bjelica et al. [25].

Our results may partly explain these conflicting results. If we analyse the mean scores of the whole population we do not see any significant difference either with controls or with a national reference population (Table 1) [19]. However, the percentage of patients with scores above the threshold for depression and general distress was significantly higher in the CMT group as compared to healthy controls collected among friends and unaffected relatives.

We then analysed factors correlating with neuropsychiatric disturbances and found that disease severity, as indicated by the CMTES, correlated with the presence of depression and general distress. Positive sensory symptoms were more frequent in anxious and distressed subjects. Overall, these data suggest that disease severity negatively affects mood and neuropsychiatric status. On the other hand, it is likely that anxious and distressed patients are more liable to perceive sensory disturbances including pain. Our results confirm the previous study by Padua and colleagues [11] in which a significant association between mood disorders and sensory symptoms was found. While in neurodegenerative disorders like Parkinson disease and Huntington’s chorea depression and general distress are symptoms of the disease itself, in CMT they are likely to be secondary to the consequences of the disease burden on patients’ life, which includes also limitations in mobility and employment. We observed no correlation with age and disease duration, suggesting that disease progression per se does not significantly affect the rate of depression and distress. It is possible that longitudinal studies may better detect a correlation with disease course, but we already know the remarkable adaptation to disability of many CMT patients as confirmed for instance by the absence of significant worsening in quality of life scales (e.g., Short-Form 36, SF36) over time [10, 26].

It is notable that a high percentage of CMT patients takes anxiolytics and/or antidepressant drugs, which again indicates that the neuropsychiatric disturbances lead many CMT patients to medical attention and to treatment. On the other hand, it is worrying that about one half of patients with depression and general distress receive no specific drug treatment at all, although we did not investigate whether psychological support was provided to them. In any case, this is a relevant finding and underlines the importance of assessing neuropsychiatric conditions in CMT patients and offering adequate treatment when needed.

Another impressive result of this study is the high percentage (70%) of patients making use of analgesic/anti-inflammatory drugs, with consumption at least twice a week in 17%. Indeed, pain is a frequent symptom in CMT, being reported by 23–85% of the patients [27] and may be skeletal-muscular or neuropathic in nature, or both. The effect of pain on mood in CMT is poorly investigated, but it is interesting to note that depressed and distressed patients reported more frequently positive sensory symptoms including pain and that consumption of analgesic/anti-inflammatory drugs was significantly higher in patients reporting such symptoms.

This study has some limitations. First, patients joining the registry and filling out the questionnaires may not fully represent the whole CMT population but those more liable to participate in investigations and followed at some of the tertiary centres. Second, we could not directly evaluate the patients with respect to neuropsychiatric disorders and we know that the HADS is insufficient to diagnose depression or anxiety disorders. However, we used higher cut-off values, those with the best sensibility/specificity ratio and positive predictive values, to rule out false positives [18]. It is worth noting that the present study was conducted before the COVID-19 pandemic and was not affected by the psychological impact of the pandemic, which led to an increase in anxiety, depression, and feelings of distress in the entire population. A third issue is the limited number of healthy controls, recruited in seven of the nine centres in a 17–37% ratio with respect to patients; while we could compare the mean HADS values of CMT patients with the general Italian population, we could rely only on our control sample for comparing percentages of subjects with scores above threshold, as this information was not available for the Italian population [19]. Further studies are needed to confirm our observation of higher rates of depressed and distressed subjects among CMT, but in any case, it is important to identify depressed/distressed patients and treat them appropriately.

In conclusion, our findings confirm that, although neuropsychiatric disorders in CMT have a frequency lower than other chronic neurological disorders such as Parkinson’s disease and multiple sclerosis, they are nonetheless an important issue that requires proper attention. CMT severity appears to be one of the determinants of depression and distress. Although depressed/distressed patients took drugs acting on anxiety and mood more frequently than controls, still one half of them is not treated. Taking care of psychological status must become part of the general assessment of CMT patients and, when needed, proper treatment administration may alleviate their disease burden.

## Supplementary Information

Below is the link to the electronic supplementary material.

Supplementary file1 (DOCX 19 KB)
