# Supplementary material for: Anxiety and depression in Charcot-Marie-Tooth disease: data from the Italian CMT national registry
Source: J Neurol. 2022 Sep 16;270(1):394–401. doi: 10.1007/s00415-022-11365-8 (PMC9483245; doi:10.1007/s00415-022-11365-8)
Supplement: Supplementary file 1 — Supplementary file1 (DOCX 19 KB) [file 415_2022_11365_MOESM1_ESM.docx]

**Supplementary table 1. Clinical characteristics of CMT patients**

|  | **Total (n=252)** |
| --- | --- |
| **Disease duration, mean ± SD** | 24.5 ± 14.8 |
| **Scoliosis, n (%)** | 45 (17%) |
| **Walking difficulties, n (%)** | 188 (74%) |
| **Orthotics aid users, n (%)** | 96 (38%) |
| **Walking support need, n (%)** | 32 (12%) |
| **Wheelchair users, n (%)** | 11 (4%) |
| **Positive sensory symptoms, n (%)** | 98 (38%) |
| **Difficulties with buttons, n (%)** | 148 (58%) |
| **Difficulties with eating utensils, n (%)** | 33 (13%) |
| **Anxiolytics/antidepressant users, n (%)**† | 45/234 (19.2%) |
| **Analgesics/anti-inflammatory users, (%)**† | 164/234 (70.1%) |

A=Anxiety; D=Depression; CMTES=Charcot-Marie-Tooth Examination Score; HADS=Hospital Anxiety and Depression Scale; SD=Standard Deviation; T=Total.

† The sum does not add up to the total because of some missing values

**Appendix.** Other members of the Italian CMT Network.

| **Name** | **Location** | **Role** | **Contribution** |
| --- | --- | --- | --- |
| Giulia Schirinzi | Fondazione IRCCS Istituto Neurologico Carlo Besta, Milan | Clinical assessor Physiotherapist | Data collection |
| Maria Montesano | Fondazione IRCCS Istituto Neurologico Carlo Besta, Milan | Clinical assessor Physiotherapist | Data collection |
| Sara Nuzzo | Fondazione IRCCS Istituto Neurologico Carlo Besta, Milan | Administrative person | Study organization |
| Francesca Oggiano | Fondazione IRCCS Istituto Neurologico Carlo Besta, Milan | Administrative person | Study organization |
| Chiara Gemelli | University of Genoa | Clinical assessor | Data collection |
| Marina Scarlato | IRCCS Ospedale San Raffaele, Milan | Clinical assessor | Acquisition of data |
| Emanuele Spina | University Federico II of Naples | Clinical assessor | Data collection |
| Maria Longo | University of Messina | Psychologist | Data collection |
